# Brain-derived neurotrophic factor (BDNF) in chronic pain research: A decade of bibliometric analysis and network visualization

**DOI:** 10.3934/Neuroscience.2024001

**Published:** 2024-01-11

**Authors:** Che Aishah Nazariah Ismail, Rahimah Zakaria, Khairunnuur Fairuz Azman, Nazlahshaniza Shafin, Noor Azlina Abu Bakar

**Affiliations:** 1 Department of Physiology, Universiti Sains Malaysia Health Campus, 16150 Kubang Kerian, Kelantan, MALAYSIA; 2 Brain and Behaviour Cluster, School of Medical Sciences, Universiti Sains Malaysia Health Campus, 16150 Kubang Kerian, Kelantan, MALAYSIA; 3 Faculty of Medicine, Universiti Sultan Zainal Abidin Medical Campus, Jalan Mahmud, 20400 Kuala Terengganu, Terengganu, MALAYSIA

**Keywords:** brain-derived neurotrophic factor, chronic pain, bibliometric analysis, VOSViewer, Harzing's Publish or Perish

## Abstract

Chronic pain research, with a specific focus on the brain-derived neurotrophic factor (BDNF), has made impressive progress in the past decade, as evident in the improved research quality and increased publications. To better understand this evolving landscape, a quantitative approach is needed. The main aim of this study is to identify the hotspots and trends of BDNF in chronic pain research. We screened relevant publications from 2013 to 2022 in the Scopus database using specific search subject terms. A total of 401 documents were selected for further analysis. We utilized several tools, including Microsoft Excel, Harzing's Publish or Perish, and VOSViewer, to perform a frequency analysis, citation metrics, and visualization, respectively. Key indicators that were examined included publication growth, keyword analyses, topmost influential articles and journals, networking by countries and co-citation of cited references. Notably, there was a persistent publication growth between 2015 and 2021. “Neuropathic pain” emerged as a prominent keyword in 2018, alongside “microglia” and “depression”. The journal Pain® was the most impactful journal that published BDNF and chronic pain research, while the most influential publications came from open-access reviews and original articles. China was the leading contributor, followed by the United States (US), and maintained a leadership position in the total number of publications and collaborations. In conclusion, this study provides a comprehensive list of the most influential publications on BDNF in chronic pain research, thereby aiding in the understanding of academic concerns, research hotspots, and global trends in this specialized field.

## 1. Introduction

Chronic pain is defined as pain lasting for more than three months or pain that exceeds the expected healing duration [1]. This pathological condition imposes a substantial burden on an individuals' quality of life, productivity, and economic well-being, and is often compounded by comorbid psychiatric conditions and substance abuse disorders [2]. The magnitude of this problem is exemplified in the National Health Interview Survey (NHIS) Longitudinal Cohort (2019–2020), which reported that nearly two-thirds (61.4%) of adults suffer from chronic pain. In the United States (US), the incidence of chronic pain (52.4 cases per 1000 person-year (PY)) surpasses that of prevalent diseases such as diabetes (7.1 cases per 1000 PY), depression (15.9 cases per 1000 PY) and hypertension (45.3 cases per PY) [3]. The early management of chronic pain and any related disabilities hinges on a comprehensive understanding of its underlying pathogenesis. Despite historical attempts to alleviate pain, including opium prescriptions in the 1600s and the use of morphine and heroin in the 1900s, chronic pain remains one of the most challenging conditions to treat [4]. However, promising prospects for chronic pain management have emerged with technological advancements.

Researchers have recently turned their attention to the role of the brain-derived neurotrophic factor (BDNF), which is a pivotal biological compound in the development of pathological pain [5]. BDNF is one of the neurotrophins that support neuronal differentiation, maturation and survival, as well as modulates neurotransmission and influences neuronal plasticity critical for learning and memory [6]. Notably, decreased BDNF levels are associated with neuronal loss in several neurodegenerative diseases, including Parkinson's disease, Alzheimer's disease, multiple sclerosis, and Huntington's disease [6]. Furthermore, multiple pre-clinical studies have reported increased BDNF levels in chronic pain conditions [7]–[9], thereby raising the possibility that excessive BDNF release contributes to chronic pain development.

In general, BDNF may act as either a pain mediator that facilitates the development of pain or as a pain modulator that regulates pain. Since the spinal cord dorsal horn is the essential gateway for the transmission of pain from the peripheral region to the brain, pain-related neuropeptides and neurotransmitters are released in this region [10]. Pre-clinical research employing models of peripheral nerve injury has demonstrated that BDNF synthesized by the spinal cord dorsal horn neurons causes neuronal hyperexcitation, which further results in pain hypersensitivity [7],[11]–[14]. Additionally, previous research has demonstrated that the release of BDNF has increased excitatory synaptic drive to excitatory neurons and decreased the synaptic drive to inhibitory interneurons in the substantia gelatinosa of the spinal cord. BDNF mediates these pro-nociceptive effects through its binding on two types of receptors: p75 neurotrophin (pain-selective p75 neurotrophin receptor) and tropomyosin receptor kinase B (also known as tyrosine receptor kinase, TrkB) [15]. However, activation of the spinal BDNF/TrkB pathway has been demonstrated to extensively contribute to the pathological mechanisms leading to neuropathic pain [16]. The activation of TrkB receptors leads to the downstream activation of various signaling pathways, including nuclear factor kappa B (NF-kB) and mitogen-activated protein kinases (MAPK). Consequently, these activated mechanisms lead to the further activation of p38, Jun N-terminal kinase (JNK), and extracellular signal-regulated protein kinase (ERK), which eventually leads to allodynia, thermal hyperalgesia, nerve injury-induced neuroinflammation, and neuropathic pain [13],[15]. In response to these intriguing findings, an array of therapeutics—ranging from chemical compounds to natural products—have been identified for their potential to effectively modulate BDNF levels. This endeavor paves the way for innovative chronic pain management strategies.

As a powerful tool, bibliometrics offers a means to describe and analyze the dynamic growth of certain research subjects. With the aid of developed software, it can visually analyze documents in a succinct and clear knowledge map [17]. This method allows researchers to comprehensively understand the status, current hotspots and future trends of certain research areas, which transcends the limitations of time and space [18]. Though numerous bibliometric studies have focused on BDNF in neurological and neuropsychiatric disorders such as depression [17], schizophrenia [19], Alzheimer's disease [20], genetic BDNF Val66Met polymorphism [21], and cognitive aging [22], there is currently a noticeable gap in the literature regarding a bibliometric analysis of BDNF's role in chronic pain. This study aims to fill this void by elucidating the hotspots and trends of BDNF in chronic pain research over the decade spanning from 2013 to 2022. It is anticipated that this analysis will provide researchers with new insights into the academic framework underpinning BDNF's role in chronic pain research and will aid in the formulation of future scientific work.

## 2. Materials and methods

### 2.1. Design and Search Strategy

The bibliometric analysis was performed by extracting data from the Scopus database on a single day (1^st^ August 2023) to avoid data inaccuracy during the analysis. This database was opted for due to its complete compilation of the global scientific research output. Moreover, the Scopus database is known for its coverage of a larger number of publications with more citations [23],[24]. The search terms used are as follows: “Brain*derived neurotrophic factor” OR “BDNF” AND “chronic pain” OR “chronic nociception” OR “chronic primary pain” OR “chronic cancer pain” OR “chronic headache pain” or “chronic orofacial pain” OR “chronic visceral pain” OR “chronic musculoskeletal pain” OR “neuropathic pain” OR “chronic neuropathic pain” AND NOT “acute pain” OR “acute nociception” OR “physiological pain”. The documents published before 2013 and after 2022 were excluded, and all the document types except retracted (n = 0) and erratum (n = 1) publications were included in this analysis. Additionally, the self-screening on the title and abstract of the documents unrelated to the core of the search query was carried out and resulted in six publications being removed.

### 2.2. Data Extraction

**Figure 1.**
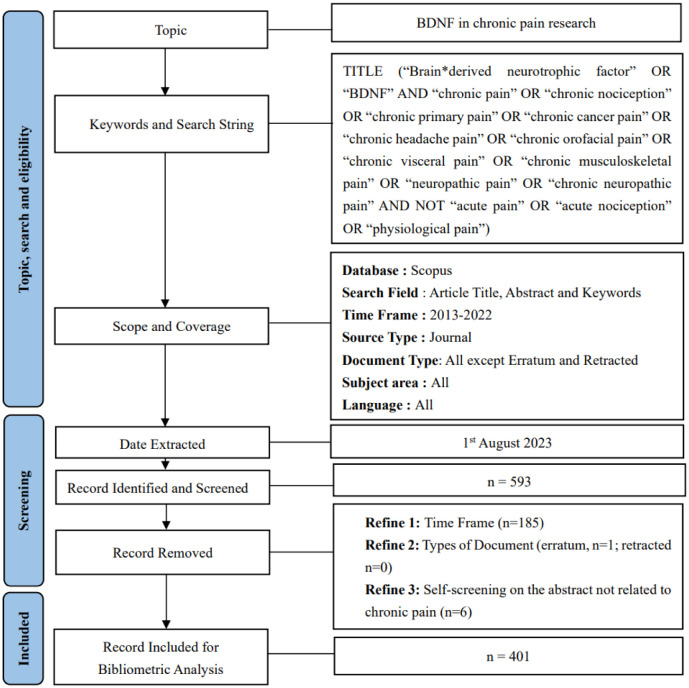
Flow diagram of the search strategy for BDNF in chronic pain research between 2013 to 2022.

The literature selection and data extraction were conducted by two independent researchers to guarantee the accuracy of the results. A total of 593 documents were extracted from the Scopus database in Microsoft Excel (.xlsx), Research Information Systems (.ris), and Comma-separated Values (.csv) formats for further analysis. The screening process was performed with a net yield of 401 documents. The data was further analyzed using Harzing's Publish or Perish and VOSViewer version 1.6.19 (Universiteit Leiden, Netherlands) in “.ris” and “.csv” formats, respectively. Figure 1 demonstrates the screening flowchart of the documents. Microsoft Excel was employed to calculate the percentages of the published documents and generate the related graphs. Harzing's Publish or Perish was applied to calculate the citation metrics, while VOSViewer was used to visualize the bibliometric networks.

## 3. Results

### 3.1. Description of the Retrieved Documents

**Figure 2.**
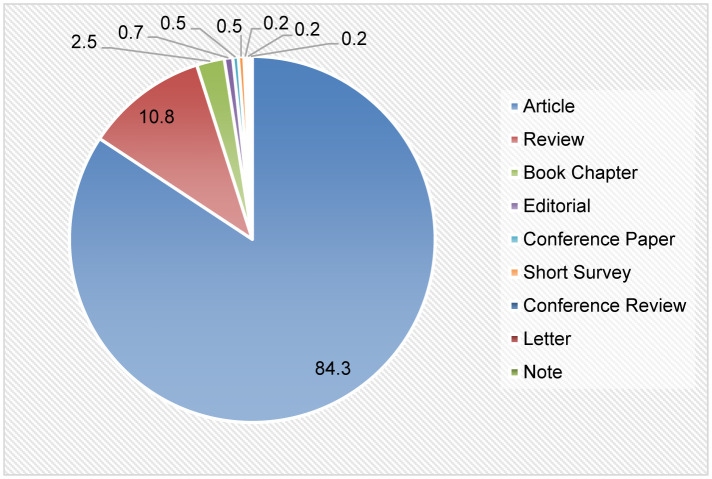
The pie chart shows the types of documents retrieved from 2013 to 2022. Articles (i.e. original work and reviews) are the highest types of documents published for BDNF and chronic pain research.

A total of 401 screened documents are derived from articles, reviews, book chapters, editorials, conference papers, short surveys, conference reviews, letters, and notes. Original articles accounted for the highest type of publication (n = 343, 85.5%), followed by several types of review articles (n = 42, 10.5%) and book chapters (n = 10, 2.49%). Meanwhile, editorials, conference papers, short surveys (n = 2, 0.5% each), letters, and notes (n = 1, 0.25% each) were the types of documents which contributed less than 1% of the overall publications (Figure 2). Meanwhile, 12 documents were written by a single author (2.99% of contribution), while the largest number of authors in a document was 24 authors. One document derived from a conference review containing no author was documented (Table 1). Besides that, the retrieved documents received a total of 9421 citations, 942.1 cites per year, 23.49 cites per paper with an h- and g-index of 50 and 73, respectively. The majority of the retrieved documents were published in English (n = 387, 96.5%), followed by Chinese (n = 10, 2.49%), Russian (n = 2, 0.5%), French and Polish (n = 1, 0.25% each).

**Table 1.** Number of authors per publication.

**Authors Per Paper**	**Total Document**	**Percentage of Contribution (%)**
0^a^	1	0.25
1	12	2.99
2	25	6.23
3	28	6.98
4	54	13.47
5	38	9.48
6	41	10.22
7	45	11.22
8	38	9.48
9	37	9.23
10	29	7.23
11	14	3.49
12	10	2.49
13	8	2.00
14	4	1.00
15	1	0.25
16	3	0.75
17	2	0.50
18	3	0.75
19	2	0.50
20	3	0.75
21	1	0.25
22	1	0.25
24	1	0.25

**Grand Total**	**401**	**100**

^a^Conference review document. No author is listed.

### 3.2. Growth of Publications and Citations

**Figure 3.**
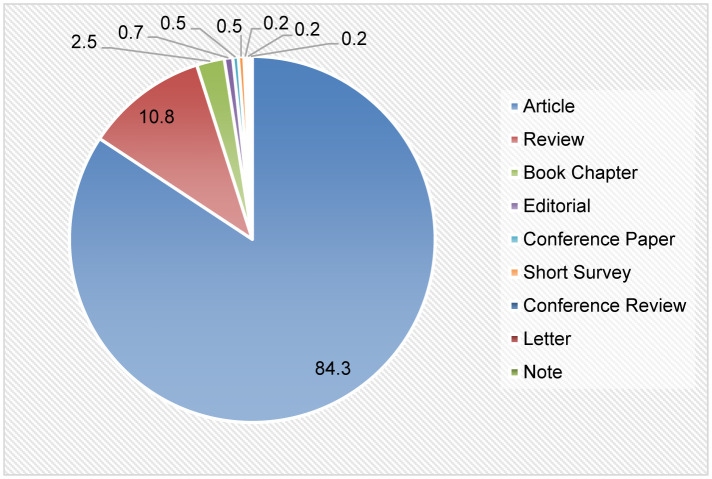
Growth of publications from 2013 to 2022 for BDNF research in the chronic pain area.

Figure 3 depicts the annual publications and citations of BDNF and chronic pain literature. A prominent fluctuation in the total publications was observed within the 10 years of the analysis. The research on chronic pain involving BDNF slightly increased in 2014 (37, 9.23%), before decreasing in 2015 (30, 7.48%). However, the total publications were progressively elevated and peaked in 2021 (56, 13.97%), before slightly dropping in 2022. In contrast, the annual citations were at the highest in 2013 (1586 total citations) and gradually declined thereafter. Among the annual citations, the total of 30 publications in 2013 were fully cited, with 54.69 citations per cited paper (Figure 3 and Table 2).

**Table 2.** Number of publications per year and citation metrics.

**Year**	**TP**	**%**	**NCP**	**TC**	**C/P**	**C/CP**	**h**	**g**
2013	29	7.23	29	1586	54.69	54.69	20	29
2014	37	9.23	36	1557	42.08	43.25	24	37
2015	30	7.48	29	1276	42.53	44	21	30
2016	33	8.23	32	983	29.79	30.72	21	31
2017	35	8.73	34	1087	31.06	31.97	18	32
2018	41	10.22	39	789	19.24	20.23	17	27
2019	42	10.47	41	923	21.98	22.51	19	28
2020	53	13.22	50	662	12.49	13.24	15	22
2021	56	13.97	50	407	7.27	8.14	11	17
2022	45	11.22	35	151	3.36	4.31	7	9

**Abbreviation:** TP, Total publication; %, annual percentage of the publications; NCP, number of cited papers; TC, total citations; C/P, average citations per publication; C/CP, average citations per cited publication; h, h-index, and g, g-index.

### 3.3. Author Keywords Co-occurrence Analysis

**Figure 4.**
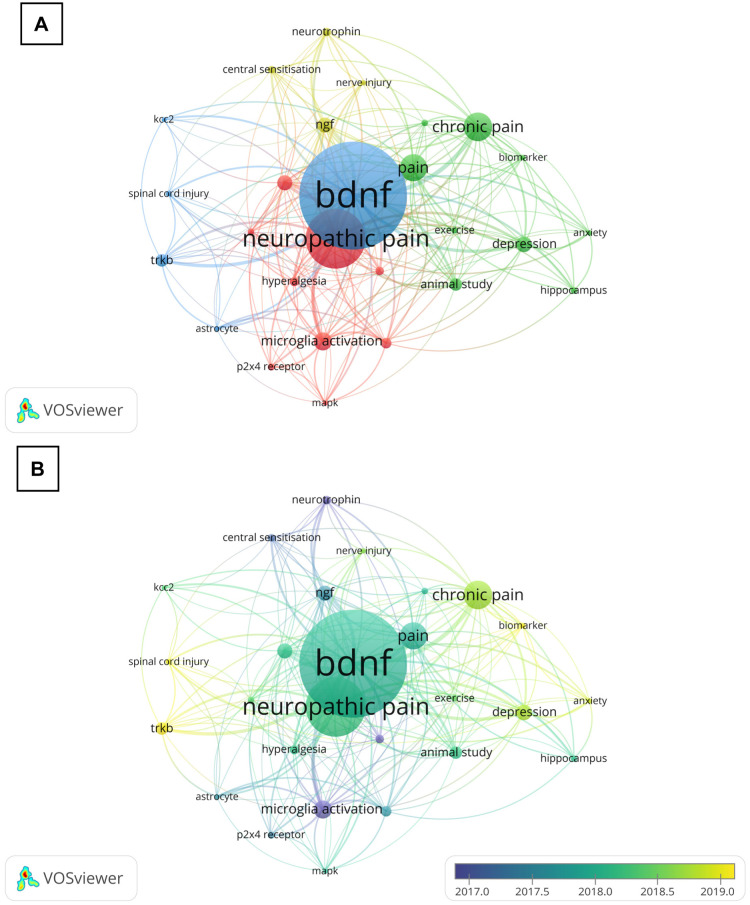
Co-occurrence of (A) Network visualisation and (B) overlay visualisation of all author keywords with a minimum of ten occurrences in the present analysis. 27 out of 725 keywords met the criterion. Apart from the core keywords, neuropathic pain shows among the highest number of occurrences which received the researchers' attention in the year 2018.

The visualization by VOSViewer software on the author keywords was conducted with a minimum of 10 occurrences. Out of 725 keywords, 27 keywords met this requirement. The most occurrences of author keywords, after excluding the core keywords related to the search query, were neuropathic pain (110), microglia activation (34), depression (29), nerve growth factor (28), spinal cord (28), animal study (23), TrkB (23), and inflammation (20) (Figure 4A). In Figure 4A, the larger circular node indicates higher occurrences of keywords being used by the authors, thereby representing the increased hotspots in this research area. The lines of the nodes denote the strength of association: the thicker the line, the higher frequency of the author's keywords appearing together in the same document. Meanwhile, the different color nodes symbolize different clusters, namely research topics [17].

According to our analysis, the author's keywords network contains four separate cluster sizes. The red cluster (cluster 1, 9 items) consists of the keywords allodynia, dorsal root ganglia, hyperalgesia, inflammation, mitogen-activated protein kinase (MAPK), microglia activation, neuropathic pain, P2X4 receptor, and spinal cord, which are related to the topic of an inflammatory-associated pain signaling mechanism. The green cluster (cluster 2, 9 items) contains animal study, anxiety, biomarker, chronic pain, cytokine, depression, hippocampus, and pain, which are mainly described topics related to psychological-associated pain. Meanwhile, the blue cluster (cluster 3, 5 items) includes astrocytes, BDNF, KCC2, spinal cord injury, and TrkB, with the topic of BDNF signaling mechanism in the pathological state. Lastly, the keywords clustered in the yellow area (cluster 4, 4 items) comprise central sensitization, nerve injury, neurotrophin, and NGF, which are related to the topic of neuronal plasticity (Figure 4A). When the further analysis explored the trends of the author's keywords by time, it was clear that the investigations focused on “microglia activation”, “dorsal root ganglia”, “neurotrophin”, and “central sensitisation” during the year 2017. During the year 2019, the majority of the research shifts to the terms “TrkB”, “biomarker”, “spinal cord injury”, and “anxiety” (Figure 4B).

### 3.4. Most Influential Works

The most influential research work was determined by a minimum number of 10 citations of a publication, which resulted in 235 documents. Table 3 lists the 10 articles that are mostly cited for BDNF and chronic pain. The narrative review article published in the Neural Plasticity journal by Ferrini and De Koninck [25] was the topmost cited, with a total of 231 citations. This is followed by the original article by Liu and colleagues [26] published in the Journal of Neuroscience, with a total of 209 citations. The third most popular article was by Gomes et al. [27], with a total of 158 citations, which is the second original experiment conducted on the rat model.

### 3.5. Journals with Topmost Publications

With a minimum of five publications and one citation, the journals with the highest publications related to BDNF and chronic research were Pain (13 documents and 474 citations) and Scientific Reports (13 documents and 265 citations). Neuroscience Letters published 12 related documents with 359 citations, followed by the Journal of Neuroscience and Molecular Pain with 9 publications (769 and 319 citations, respectively). Meanwhile, the Journal of Pain Research, Brain Research Bulletin, Experimental Neurology, Journal of Pain, Molecular Neurobiology, and PloS One published 6 documents with different numbers of total citations (range: 93–247 citations) (Table 4).

### 3.6. Bibliographic Coupling by Country

**Figure 5.**
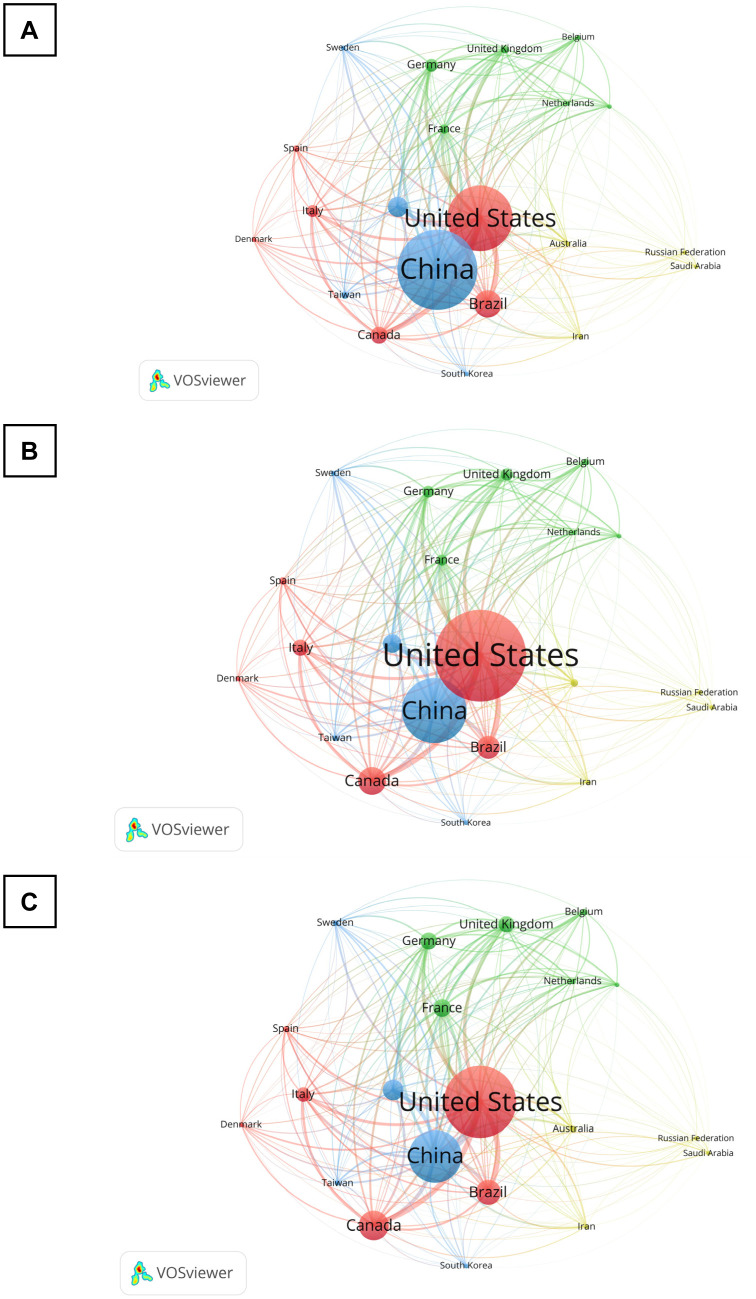
Bibliographic coupling by country. The network visualizations portray the (A) total documents, (B) total citations, and (C) total link strength of the publications in BDNF and chronic pain research between the countries.

Figure 5 shows the total number of documents, citations, and link strengths for the research of BDNF and chronic pain between countries. The analysis includes the countries with at least five publications and 10 total citations. The results revealed that 11 countries met the thresholds out of 49 countries. In terms of the total number of documents, China published the largest number of documents (132), followed by the US (108), as proven by the larger size of the circles (Figure 5A). There was a huge gap in the number of documents from the other countries, as Japan published 45 documents, Canada produced 33 publications, Germany published 21 documents, and Italy published 20 documents. However, the total citations were the highest in the documents published within the US as compared to China (3468 vs 2454 total citations, respectively) (Figure 5B). In terms of collaborations, as indicated by total link strength (TLS), the US received the highest number of collaborations (27,097 TLS), followed by China (19,823 TLS) and Canada (11,167 TLS) (Figure 5C).

**Table 3.** Ten most influential articles.

**No**	**Authors**	**Article title**	**Source**	**Year**	**TC**	**C/Y**	**Reference**
1	Ferrini, F., De Koninck, Y.	Microglia control neuronal network excitability via BDNF signalling	Neural Plasticity	2013	231	23.1	[25]
2	Liu, Y., Zhou, L. -J., Wang, J., Li, D., Ren, W. -J., Peng, J., Wei, X., Xu, T., Xin, W. -J., Pang, R. -P., Li, Y. -Y., Qin, Z. -H., Murugan, M., Mattson, M. P., Wu, L. -J., Liu, X. -G.	TNF-α Differentially Regulates Synaptic Plasticity in the Hippocampus and Spinal Cord by Microglia-Dependent Mechanisms after Peripheral Nerve Injury	Journal of Neuroscience	2017	209	34.83	[26]
3	Gomes, C., Ferreira, R., George, J., Sanches, R., Rodriguez, D. I., Gonçalves, N., Cunha, R. A.	Activation of microglial cells triggers a release of brain-derived neurotrophic factor (BDNF) inducing their proliferation in an adenosine A2A receptor-dependent manner: A2A receptor blockade prevents BDNF release and proliferation of microglia	Journal of Neuroinflammation	2013	158	15.8	[27]
4	Taves, S., Berta, T., Chen, G., Ji, R. -R.	Microglia and spinal cord synaptic plasticity in persistent pain	Neural Plasticity	2013	145	14.5	[28]
5	Yalcin, I., Barthas, F., Barrot, M.	Emotional consequences of neuropathic pain: Insight from preclinical studies	Neuroscience and Biobehavioural Reviews	2014	142	15.78	[29]
6	Taylor, A. M. W., Castonguay, A., Taylor, A. J., Murphy, N. P., Ghogha, A., Cook, C., Xue, L., Olmstead, M. C., De Koninck, Y., Evans, C. J., Cahill, C. M.	Microglia disrupt mesolimbic reward circuitry in chronic pain	Journal of Neuroscience	2015	132	16.5	[30]
7	Khan, N., Smith, M. T.	Neurotrophins and neuropathic pain: Role in pathobiology	Molecules	2015	129	16.13	[30]
8	Nijs, J., Meeus, M., Versijpt, J., Moens, M., Bos, I., Knaepen, K., Meeusen, R.	Brain-derived neurotrophic factor as a driving force behind neuroplasticity in neuropathic and central sensitization pain: A new therapeutic target?	Expert Opinion on Therapeutic Targets	2015	128	16	[32]
9	Zhou, L. -J., Peng, J., Xu, Y. -N., Zeng, W. -J., Zhang, J., Wei, X., Mai, C. -L., Lin, Z. -J., Liu, Y., Murugan, M., Eyo, U. B., Umpierre, A. D., Xin, W. -J., Chen, T., Li, M., Wang, H., Richardson, J. R., Tan, Z., Liu, X. -G., Wu, L. -J.	Microglia are indispensable for synaptic plasticity in the spinal dorsal horn and chronic pain	Cell Reports	2019	119	29.75	[33]
10	Richner, M., Ulrichsen, M., Elqmegaard, S. L., Dieu, R., Pallesen, L. T., Vaegter, C. B.	Peripheral nerve injury modulates neurotrophin signaling in the peripheral and central nervous system	Molecular Neurobiology	2014	114	12.67	[34]

**Abbreviations:** TC, Total citations; and C/Y, citations per year.

**Table 4.** Eleven active journals.

**Source Title**	**TP**	**TC**	**Publisher**	**CS 2022**	**SJR 2022**	**SNIP 2022**
Pain	13	474	Lippincott Williams and Wilkins	12.5	2.445	3.151
Scientific Reports	13	265	Nature Publishing Groups	7.5	0.973	1.312
Neuroscience Letters	12	359	Elsevier Ireland Ltd	5.9	0.802	0.777
Journal of Neuroscience	9	769	Society for Neuroscience	9.9	2.35	1.471
Molecular Pain	9	319	BioMed Central Ltd and SAGE Publications Inc.	5.7	0.824	0.866
Journal of Pain Research	8	150	Dove Medical Press Ltd	4.8	0.667	1.083
Molecular Neurobiology	6	247	Humana Press and Springer	10.7	1.325	1.132
PloS ONE	6	231	Public Library of Science	6	0.885	1.253
Experimental Neurology	6	217	Academic Press Inc.	9.1	1.38	1.135
Journal of Pain	6	183	Churchill Livingstone Inc. and Elsevier B.V.	7.9	1.363	1.794
Brain Research Bulletin	6	93	Elsevier Inc.	6.7	0.885	0.861

**Abbreviations:** TP, Total publications; TC, Total citations; CS, CiteScore; SJR, SCImago Journal Rank; SNIP, Source Normalised Impact Per Paper.

### 3.7. Co-citation Analysis by Cited References

**Figure 6.**
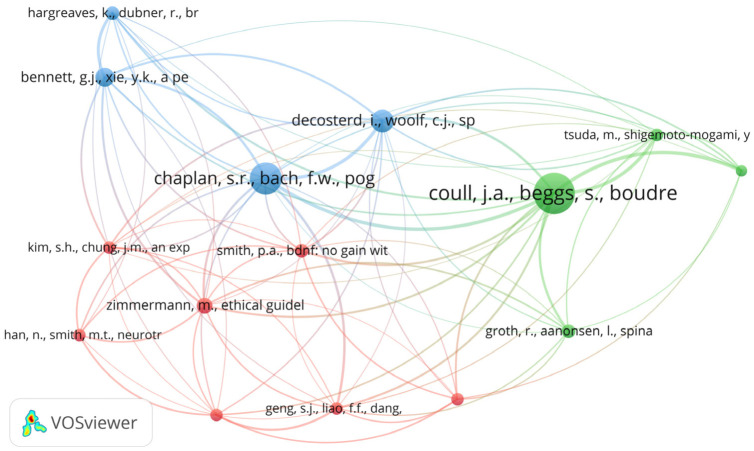
Co-citation analysis of cited references.

This analysis was conducted to track the pairs of documents that are cited together by the other researchers in the references. The VOSviewer software was employed to generate the co-citation by a cited references map, where the fractional counting approach was selected, with a minimum of 10 citations of a cited reference. Out of 24,206 cited references, 15 of the cited references met the threshold and were further grouped into three cluster networks (Figure 6). The red cluster consists of seven articles, which share the theme of chronic pain models. The green cluster consists of four articles, which are related to the BDNF-related signaling mechanism to neuropathic pain development. Meanwhile, the blue cluster represents four documents, which are related to the protocol of chronic pain assessment in animal models (Figure 6 and Table 5). In specific, the original article written by Coull et al. [14] was the most influential work, as indicated by the highest TLS (30), the highest number of total citations (36) and the highest number of links (13). It is followed by three other original articles authored by Chaplan et al. [35], Decostered and Woolf [36], and Bennett and Xie [37] (Table 6).

## 4. Discussion

To the best of our knowledge, this article is the first to use the bibliometric technique to present BDNF in the context of chronic pain research. Based on the documents retrieved from the Scopus database, a total of 401 documents were identified in the decade of research from 2013 to 2022. The majority of documents were from primary research and report data from preclinical or clinical studies. The document with the largest number of authors was a clinical trial conducted by Ribeiro et al. [38] that had 24 authors. The majority of the published documents (i.e., 54 documents) listed four authors per paper.

Over the years, academic interest in the relationship between BDNF and chronic pain has grown tremendously. This is shown in the annual increase of total publications within a decade of research, with the highest publication number reported in 2021. Most of the documents published that year were related to neuropathic pain and depression; in a similar manner, a peak of publications that linked BDNF to depression was reported in 2021 by a bibliometric analysis by He et al. [17]. Previous studies highlighted the crucial association between pain and depression through the changes in the BDNF-TrkB receptor signaling mechanism [39]. Therefore, the trends of BDNF in depression and chronic pain research may be interconnected. Similarly, microglia has gained the researcher's focus for a few decades due to its promising prospect in combating neuropathic pain. Zhang et al. [40] conducted a bibliometric study to comprehensively evaluate microglia-associated neuropathic pain research between the years 2000 to 2021. The annual increase in the total number of publications and citations of this research area, which peaked in the year 2021, possibly explained the similar increased trends of BDNF in chronic pain research, as BDNF is closely related to the mechanism of P2X4 receptor-induced microglia activation during the pathogenesis of neuropathic pain [41],[42]. In contrast, the trend of total citations of BDNF in chronic pain research annually declined within the 10 years of the analysis. The total number of citations quantifies the overall impact of specific research documents and represents the quality of the research [43],[44]. The decreasing trend of BDNF in chronic pain research could be due to the time factor. Citations gradually accumulate for articles, meaning that older articles have a greater opportunity to be cited and, all other things being equal, should possess more citations compared to newer articles [45],[46].

The core and essence of a publication are condensed in its keywords. The keyword co-occurrence analysis indicates the research hotspots and reflects the development of a knowledge area. In this prospect, the keyword “neuropathic pain” was at the topmost selected author's keywords and became the center of the researchers' attention, especially in the year 2018. Based on the bibliometric analysis of neuropathic pain starting in the year 2010, researchers began to extend their investigations to various treatments and paid closer attention to prognostic evaluations, cutting-edge therapeutic approaches, quality of life, and other facets of neuropathic pain therapy [47]. Additionally, this analysis indicates that neuropathic pain remains a pathological condition that needs to be urgently solved; researchers should focus on developing new therapeutics, understanding the pathological mechanism, and strengthening the pain management strategy which relates to BDNF.

Meanwhile, the most active journal based on the number of published documents in the context of BDNF and chronic pain was Pain®, followed by Scientific Reports and Neuroscience Letters. The Pain® journal is a leading journal devoted to pain medicine and research, with a high impact factor and rank (IF: 7.926 and ranked as Q1 journal in the Web of Science database). This journal produced its first issue in March 1975 and will be celebrating its 50-year establishment in 2024. In the year 2022, this journal was ranked as the fifth leading journal under the category of Anaesthesiology, 25th under Clinical Neurology, and 33rd under Neuroscience categories. This is a very established and impactful journal with the highest CiteScore, SJR and SNIP in the year 2022. Apart from that, the highly cited publication indicates the increased influence or impact of a research work. In the context of BDNF in chronic pain research, a review written by Ferrini and De Koninck [25] published in Neural Plasticity received the highest attention from researchers. This narrative review described the involvement of BDNF via TrkB-mediated signaling in the microglia-neuron crosstalk that contributes to the occurrence of neuronal excitability. The second and third most influential publications were the in-vivo and in-vitro studies authored by Liu et al. [26] and Gomes et al. [27], respectively, which investigated a similar research focus as Ferrini and De Koninck [25] (i.e., related to the microglial induced-BDNF mechanisms during neurodegenerative pathologies). These three documents are freely accessible to the readers. Thus, it can be deduced that well-deliverables, freely available, and the popularity of the discussed topic of the articles during those publication years could be the key factors to the increase in the total number of citations of a document. However, it is undeniable that other similar studies were also impactful, though they possibly differ in the aforementioned factors. In fact, the total number of citations does not fully reflect the impact of a published document, since many factors contribute to this condition [48]. The publication date, research topic, and document type are among the variables that may have an impact on the citation analysis. Therefore, erroneous, inaccurate, or overstated citation counts may be detected in the citation analyses. Furthermore, the majority of the journals apply a pay-per-view approach rather than offering open access to the publication. Since not everyone can access certain documents, this factor may not only implicate the total citation of a document, but also its growth [47].

According to the bibliographic coupling by country, it was discovered that China produced the highest number of publications within the 10 years of chronic pain research related to BDNF. In China, chronic pain is a major health issue and this country has the largest geriatric population in the world. There was a high incidence of chronic pain in the elderly, especially in those that were 80 years old [49]. Unfortunately, the elderly sought out less pain-related health care due to their varied cultural perception and economic background. Thus, it is possible that the increased number of investigations on either the effects of certain medicinal or herbal treatments on BDNF in the aspect of chronic pain was conducted by local researchers to solve their country's biggest health problem. Notably, the US possesses the most influential works, as indicated by its highest total number of citations of documents. The bibliographic coupling strength of a country increases with the increased number of references or links they have in common [50]. In addition, this country has the largest collaboration, especially with Canada, Brazil, Germany, France, United Kingdom, and Italy. As the world's preeminent nation in the medical sector throughout the past few decades, the US is well renowned for having the most influence and quality of literature [17]. This extensive collaboration between the different countries illustrates that this research has attracted global attention in order to understand the significance of BDNF in chronic pain.

Apart from that, a co-citation analysis involves tracking pairs of documents that are cited together in the source articles [50]. The co-citation of cited references indicates that the references cited in the two different documents are related to one another, although they are not directly citing each other. More citations of these two documents by other documents indicate stronger linkages or relationships, which indirectly creates research clusters that share some common themes. In this analysis, three cluster networks were identified from the co-citation analysis of the cited reference. The first cluster comprised the largest number of documents that shared the same theme, which described animal models of chronic pain with or without relating to BDNF signaling. The second cluster of the co-citation of cited references emphasized BDNF-related signaling in the spinal cord region in the pathogenesis of neuropathic pain. Meanwhile, the third cluster indicated different protocols or methods of chronic pain assessment, where the documents cited were among the first papers to describe the important methods of evaluating specific types of pain. Coull et al. [14] received the highest strength and total number of citations of the cited reference. This is possibly related to the important findings in the pathogenesis of neuropathic pain that caught the researcher's attention.

This bibliometric analysis has several limitations, which include potential biases such as self-citations and language bias, the dependency on research fields, time lags in data availability, the potential for manipulation and gaming, the lack of context and qualitative assessment, the difficulty of assessing individual researchers, the static nature of bibliometric data in a dynamic research landscape, its limited scope in capturing all types of scholarly outputs, and ethical concerns [51],[52]. Meanwhile, in this current study, we have encountered a limitation where the analysis related to the authors regarding either the co-authorship, citation, bibliographic coupling or co-citation of the cited authors were not easily analyzed due to the authors' similar initials, especially the researchers from China. For instance, the initial of “Zhang Y” comes from the researchers' names of Zhang, Yuangi and Zhang, Yun, who published different manuscripts on BDNF in chronic pain research. Therefore, a bibliometric analysis related to the author's name was not performed to avoid the data inaccuracy. These limitations highlight the need for a cautious and holistic approach when using bibliometrics in research evaluation, complementing it with other assessment methods to gain a more well-rounded view of the research impact and quality [58].

**Table 5.** The highest total link strength, total citations and number of links for co-citation by cited references analysis.

**Total Link Strength (TLS)**	**Total Citations**	**Number of Links**	**References**
30	36	13	Coull et al. [14]
22	28	14	Chaplan et al. [35]
16	20	13	Decostered and Woolf [36]
14	17	10	Bennett and Xie [37]
12	14	12	Zimmermann et al. [54]
17	12	9	Groth and Aanonsen [55]
7	12	9	Hargreaves et al. [56]
9	12	11	Smith [57]
11	11	12	Geng et al. [58]
6	11	7	Khan and Smith [31]
7	11	11	Kim and Chung [59]
9	11	10	Merighi et al. [60]
7	11	9	Pezet and McMahon [61]
9	11	10	Tsuda et al. [62]
9	10	7	Coull et al. [63]

**Table 6.** Summary of BDNF and chronic pain research co-citation of cited references network (Cluster 1–3).

**Cluster**	**Representative Authors**	**Content**	**Theme of Cluster**
1 (Red, 7 documents)	Geng et al. [58]	Spinal BDNF/TrkB-mediated signalling pathway is involved in nerve injury-induced neuropathic pain (mechanical allodynia) through NMDA receptor activation	Chronic pain models
	Khan and Smith [31]	Description of peripheral nerve ligation rat model to evaluate the effectiveness of NGF antagonist	
	Kim and Chung [59]	Development of experimental rat model for segmental spinal nerve ligation	
	Merighi et al. [60]	Role of BDNF in modulating pain responses including hyperalgesia and allodynia	
	Pezet and McMahon [61]	BDNF as central modulator of pain responses (allodynia and hyperalgesia) through several mechanisms of actions	
	Smith [57]	Evaluation of pain response (hyperalgesia) in relation to spinal BDNF expression	
	Zimmermann [54]	Ethical guidelines on developing chronic pain model in animals	
2 (Green, 4 documents)	Coull et al. [14]	Inhibition of spinal microglia-induced BDNF release attenuated neuropathic pain responses	Spinal BDNF-related signalling in neuropathic pain
	Coull et al. [63]	Disruption of spinal KCC2 co-transporter leads to increased neuropathic pain responses in rat	
	Groth and Aanonsen [55]	Spinal BDNF-induced hyperalgesia is associated with NMDAR activation	
	Tsuda et al. [62]	Attenuation of spinal microglia activation-induced P2X4 receptor reversed tactile allodynia in peripheral nerve injury rat	
3 (Blue, 4 documents)	Bennett and Xie [36]	Indication of pain responses (hyperalgesia, allodynia and spontaneous pain) after development of peripheral mononeuropathy by sciatic nerve ligation in rat model)	Protocol of chronic pain assessment
	Chaplan et al. [35]	Method of evaluating pain response (tactile allodynia) in spinal nerve ligation rat model	
	Decostered and Woolf [36]	Methods to develop neuropathic pain by spinal nerve ligation in rats	
	Hargreaves et al. [56]	Methods to evaluate thermal hyperalgesia in chronic pain rats (carrageenan-induced inflammation)	

## 5. Conclusion

This bibliometric analysis and visualization of BDNF in chronic pain literature from 2013 to 2022 revealed a consistent growth in research interest, with a notable upsurge in 2020 and 2021. “Neuropathic pain” emerged as a pivotal keyword in 2018, and the review authored by Ferrini and De Koninck [25] was identified as the most influential publication, while the journal “Pain” was prominently featured. China led in the number of publications, followed by the United States. A paper by Coull et al. [14] stood out with the highest total link strength and number of citations in co-citation analyses. This study provides valuable insights for future research and underscores the growing significance of BDNF in chronic pain development.

## Use of AI tools declaration

There was no Artificial Intelligence (AI) tool applied in the preparation of the manuscript.
